# The Bogazici face database: Standardized photographs of Turkish faces with supporting materials

**DOI:** 10.1371/journal.pone.0192018

**Published:** 2018-02-14

**Authors:** S. Adil Saribay, Ali Furkan Biten, Erdem Ozan Meral, Pinar Aldan, Vít Třebický, Karel Kleisner

**Affiliations:** 1 Department of Psychology, Boğaziçi University, Istanbul, Turkey; 2 Department of Computer Science, Universitat Autònoma de Barcelona, Barcelona, Spain; 3 Department of Social Psychology, Tilburg University, Tilburg, The Netherlands; 4 Department of Philosophy and History of Science, Charles University, Prague, Czech Republic; 5 National Institute of Mental Health, Klecany, Czech Republic; Bournemouth University, UNITED KINGDOM

## Abstract

Many sets of human facial photographs produced in Western cultures are available for scientific research. We report here on the development of a face database of Turkish undergraduate student targets. High-resolution standardized photographs were taken and supported by the following materials: (a) basic demographic and appearance-related information, (b) two types of landmark configurations (for Webmorph and geometric morphometrics (GM)), (c) facial width-to-height ratio (fWHR) measurement, (d) information on photography parameters, (e) perceptual norms provided by raters. We also provide various analyses and visualizations of facial variation based on rating norms using GM. Finally, we found that there is sexual dimorphism in fWHR in our sample but that this is accounted for by body mass index. We present the pattern of associations between rating norms, GM and fWHR measurements. The database and supporting materials are freely available for scientific research purposes.

## Introduction

Humans are a highly social species and possess the ability to identify each member of the species from their facial features. Faces not only allow perception of identity (i.e., who exactly a target person is) but also convey important information about emotions and intentions of the target person and such information critically helps people navigate social interactions [1]. Furthermore, the human face is thought to carry distinct cues leading perceivers to make inferences about the personality and behavioral patterns of the person whose face they are viewing [2]. Whether accurate or not, such inferences are made very rapidly [3]. However, they are not just fleeting cognitions but are tied to real-world outcomes like mate choice [4] and career success [5,6]. Inferences from faces may even affect life-and-death decisions such as the decision to impose capital punishment on a person [7–9]. In some senses a person’s face is a “window to his/her soul” [10] as the common saying goes.

The burgeoning scientific literature on the importance of faces in person perception in social psychology and related fields reflects this recognition of the centrality of faces in social perception and relationships [1,11]. While faces were relatively neglected for a good portion of the history of social psychology [12], they have received increasing attention especially since the early 2000s. Many research paradigms in the behavioral sciences, whether they are focused directly on face perception or not, employ static human facial photographs. For instance, in research on spontaneous trait inferences [13], a facial photograph is typically used to represent each “actor” whose behavior (represented in short, trait-implying sentences) is the crucial stimuli [14]. This is not absolutely required but serves to make the task more realistic and engaging for participants since faces are easily processed rich visual stimuli that uniquely identify individuals. In other research, faces take a more substantive role. For instance, many researchers are interested in the accuracy of inferences made by naive participants from faces. One example is research demonstrating that participants’ judgments of the competence of politicians’ faces were predictive of real-world election results [15]. Subsequent research extended this finding to other domains [16,17] while research in neighboring fields such as biology and anthropology focused more heavily on identifying morphological features that may aid inferential accuracy. For instance, the facial width-to-height ratio (fWHR) is an objectively measurable facial feature that is thought to be related to testosterone [18], which in turn is known to be linked with dominance and aggressiveness [19]. Thus, naive perceivers may accurately judge such nuanced traits as fighting ability [20] perhaps because they are implicitly attentive to this facial feature or other features correlated with it [21].

Since each human face is unique and complex, high-quality photographs produced under standardized conditions are required to conduct rigorous research on faces. When such images are lacking, researchers may turn to other sources such as the internet, but this may not yield optimal results. Yet, it is known that these photographs pose serious challenges for the measurement of facial features such as fWHR [22]. In fact, even when photographs are taken with appropriate equipment under standard conditions, technical aspects of the production process that are not available to immediate human perception may pose challenges to research conducted using those photographs. For instance, Třebický, Fialová, Kleisner, and Havlíček [23] showed that fWHR measurements differ significantly depending on combination of shooting distance and focal length of the camera lens used to produce the image. While these peripheral qualities of the stimuli may not always be ideal or under the researcher’s control, it is at least necessary to have detailed information about them so that the research can stay aware of the limitations imposed on the research by the use of these stimuli, as the case example of fWHR research clearly shows.

In addition, many common tasks are performed by researchers on the photographs in accordance with common trends in research content and methods. Researchers lose valuable time when they fail to combine efforts to perform these common tasks and may also create unnecessary noise and measurement error in their data by performing these tasks in unintentionally different ways. Once again, fWHR measurement may serve as an example: The exact process by which this measurement is taken—and for that matter, how photographs are produced or acquired—by different research teams is not always available in published reports. Likewise, transformations such as morphing and averaging faces, requiring the tedious task of facial landmark placement, are widely performed for research.

Finally, it is often helpful, if not critical, to have information regarding how target faces are perceived by people in general. For instance, researchers testing the effects of facial trustworthiness may seek to control for facial attractiveness as these are typically correlated [24]. However, gathering these facial ratings within a single study may not be plausible because it introduces participant fatigue or because these ratings affect (and are affected by) other steps of the study’s procedure. If the researchers wish to avoid these latter problems, then they are forced to recruit additional participants to obtain reliable ratings for multiple dimensions of face perception (e.g., trustworthiness, attractiveness, dominance, etc.)

In sum, the quality of photographs used in scientific research is directly tied to the solidity of conclusions reached in such research. For this reason, increasing the number of freely available sets of facial photographs that enable rigorous scientific research is an important goal. Freely sharing supporting materials such as facial landmarks, metrics, and norms should further facilitate scientific research by both contributing to standardization of methods and saving resources.

### The present contribution

With these in mind, in the present contribution, we sought to establish a database of faces—The Bogazici Face Database—to facilitate research using faces and to add uniquely to existing databases. Specifically, most published face sets are based on individuals residing in Western societies. Because information on facial databases is widely available across the internet (e.g., http://web.mit.edu/emeyers/www/face_databases.html) and in journal articles [25], we refrain from discussing these databases further here. Consequently, any ethnic diversity present in these sets is confined to the diversity present in Western societies. For instance, databases developed in the U.S.A. may include individuals of Asian, Hispanic, and African-American descent [25] but are unlikely to include many individuals of Mediterranean, Balkan, and Middle Eastern background. The Turkish population is a mixture of wide genetic influences [26] and ethnic backgrounds. However, to the best of our knowledge, there is no published database of Turkish faces. Thus, we sought to establish such a database from a relatively wide sample of undergraduate students. While this meant that our sample lacked diversity in terms of age, the fact that it was drawn from the largest (population-wise) and most diverse city in Turkey (Istanbul) made it highly likely that it reflected the genetic and ethnic diversity that has been present in this region for centuries (e.g., Greeks, Armenians, Jews, Kurds, Roma, peoples of the Caucasus mountains, etc.). Second, we sought to create a database that would conform to several standards, making the images suitable for rigorous scientific research. Third, we sought to provide supporting materials that should allow researchers to accurately judge the suitability of the whole set, and of individual images, for their specific purposes. This included technical details of each image, basic demographic information for targets, and appearance-related information regarding the photographs. Critically, we collected data from a large sample of raters who were similar in demographics to the targets, to establish norms for how each target face was perceived in general. We also provided materials to aid common research tasks, specifically landmarks and a currently popular facial metric.

Below we explain in detail the methods we followed to establish the database and its supporting materials, together with methods we used for preliminary statistical analysis. The database is freely available for non-commercial, research purposes (see Discussion). We refer to this resource as “The Bogazici face database,” named after the university whose students served as targets. An unrelated face database (see http://bosphorus.ee.boun.edu.tr/) was previously developed at the same university and is referred to with a similar name (i.e., “Bosphorus,” the English name for “Bogazici”).

## Method

The procedure of the current research was approved by Boğaziçi University’s Human Research Ethics Board (PEAK 2015-03-005 (collection of data from the target sample) and INAREK 2017/1 (collection of data from the norming sample).

Since our main goal was to develop a database and provide rating norms, rather than to test specific hypotheses, we aimed to simply maximize sample size for both the target faces and raters within the period of time that we deemed feasible for data collection (one academic year for target faces and three months for the norming survey).

### Data availability

All data reported in this manuscript is available (from the Department of Psychology at Bogazici University by e-mail) at no cost as part of the face database, for individuals affiliated with a research institution and who agree to terms of use.

### Target sample

Students enrolled in introductory psychology courses (which include students from a wide variety of majors) at Bogazici University, Istanbul, Turkey, were invited to take part in the development of the database via e-mail. Those who agreed to participate and completed the procedure were reimbursed with extra course credit. A total of 543 students completed the procedure, of whom 264 (149 females, 115 males; M_age_ = 21.65, SD_age_ = 1.893, age range = 19–32) granted us written permission to share their photographs with other researchers. Apart from three non-Turkish students, all indicated being Turkish nationals. Each target individual provided a signed consent form indicating their approval that their photograph can be used for scientific research purposes. Photographs were taken in 2015 and 2016.

### Equipment and setup

Photographs were taken by a professional photographer in a local photography studio near the two main campuses of Bogazici University. A Nikon D90 (APS-C) camera equipped with an Nikkor 18-105mm f/3.5–5.6 G ED AF-S DX VR Zoom lens was used for majority of the photographs. The distance between the camera and the participant was approximately 3 meters. All photographs were taken in a perpendicular angle against a white seamless background. A total of 5 flashes (Hensel ParaFlash) were used: 2 for face illumination, 2 for background, and 1 as hair rim light. The images were shot in manually set white balance using Nikon Standard Picture Control setting. The raw image file was exported to sRGB JPEG format, and digitally sent to the second author.

Some technical parameters varied between photos. Information from Exchangeable image file format (EXIF), which contains these parameters, was extracted using Exiftool (http://www.sno.phy.queensu.ca/~phil/exiftool/) and is provided in the accompanying spreadsheet (from here on referred to as “information file”). It contains the following EXIF fields: Model, LensID, ShutterSpeedValue, ApertureValue, ISO, FocalLength, FocalLengthIn35mmFormat, and SubjectDistance.

### Stimuli collection

Targets were given preliminary instructions in the invitation e-mail and requested to show up, if possible, not wearing any makeup and without facial hair. Upon arrival at the studio, further instructions were given in writing. Specifically, targets were asked to remove as much facial and head decoration (eyeglasses, earrings, piercings, etc.) as possible and to pose for the camera with neutral facial expression. A single photograph was taken using the equipment and setup explained above.

Despite efforts, many students failed to follow the instructions, resulting in photographs with makeup, hair and clothing covering parts of the face, and visible piercing. This limitation may be an advantage for researchers in need of more natural images. To make it easier for researchers to get an overview of the presence of features that may hinder scientific research, two authors coded the photographs for presence of makeup, eyeglasses, facial hair, facial decoration and accessories, obstruction of ears, emotional expression, and head position. A third author resolved inconsistencies in these codings. This information is provided for each photograph in the information file.

Before finishing the procedure, participants also reported their year of birth, gender, their nationality (Turkish or other). Self-reported height (cm) and weight (kg) were also gathered for a subset (*n* = 185) of targets.

### Stimuli standardization

**Fig 1:**
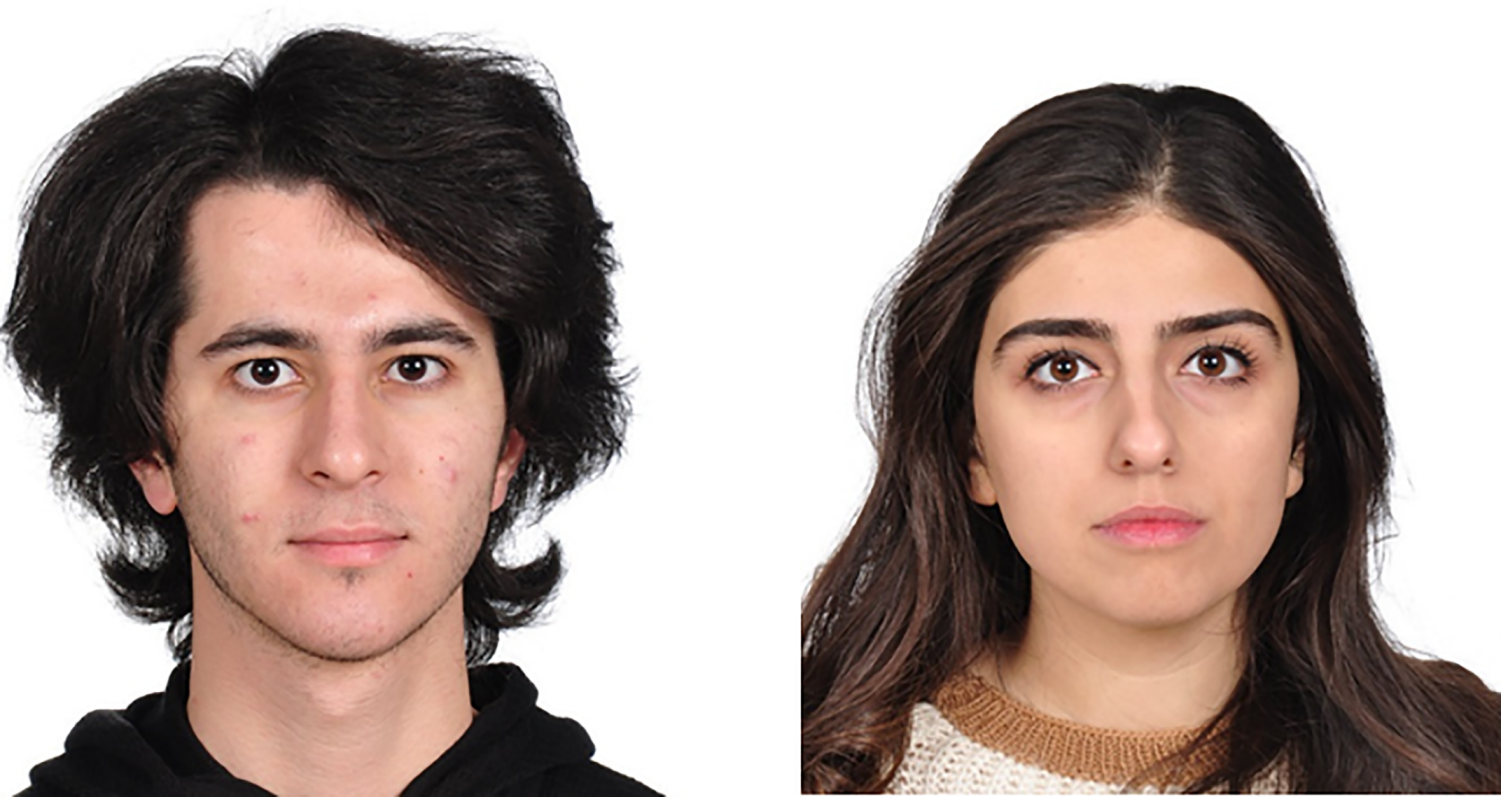
Sample facial photographs from the database. Released under a CC BY license, with permission from the face-bearers.

Standardization of photographic images was carried out using the free and open source image manipulation software GIMP (version 2.8.16) with the “export layers” add-on (version 2.4; see http://registry.gimp.org/node/28268). A single photo was randomly chosen and used as a basis for standardizing the position of all other photos in relation to the frame. Each of the remaining photos were opened as a layer onto this base photo. Using GIMP’s ruler tool, the position of the base and target photos were matched using a vertical line crossing the middle of the nose and a horizontal line crossing both irises. Consequently, when the image files are displayed on the computer screen, they have the same position, that is, the faces are aligned with each other in terms of their position within the frame. The raw image files had dimensions that were unnecessarily large for subsequent online ratings. Therefore, the images were reduced to 600 pixels (width) * 745 pixels (height) and saved in.PNG format. Two sample images are shown in Fig 1. Note that the images were not meant for color analyses as they were not color calibrated. Geometric morphometric (GM) landmarks were placed on.BMP versions of the photographs, therefore we also provide the.BMP version for each photograph.

All image files were named to start with “BFD” so that it is possible to track their source they are separated from their original folder or when multiple databases are used. Next, the letter “M” (male) or “F” (female) is used to indicate the self-reported gender of the target. Thus, the image files can easily be sorted for gender-based viewing and selection in modern computer operating systems. Alternatively, target gender is available in the information file. Last, the filename contains the unique ID number given to each photograph. This number is used consistently in the supporting information to refer to the particular photograph. The ID number is arbitrary and does not signify any feature of the image.

### Norms

We collected data from a separate sample of participants to establish norms for each facial photograph, using the standardized (see *Stimuli standardization*) images. Data were collected from adults and university undergraduates. We excluded Bogazici University students due to the possibility that students in that university could recognize the target individuals. Participants were given the chance to enter a lottery to win several online bookstore vouchers (worth approximately 31 EUR / 33 USD in April 2017) and some were additionally given extra course credit for participation. Data was collected online using Qualtrics Survey Suite.

Participants were included in the analyses even if they responded to a very small subset of items, since we were interested in the quality of faces rather than participants. There were 1207 participants (862 female, 225 male, 120 unreported; *M*_age_ = 22.09, *SD*_age_ = 3.66). The majority of participants were undergraduate students (*n* = 979), resided in the three largest cities in Turkey (440 from Istanbul, 262 from Izmir, 132 from Ankara), and reported being of Turkish ethnicity (*n* = 902). Others were Kurdish (*n* = 76) and of various other etnicities (*n* = 58) such as Armenian, Greek, Arabic, Bosnian, and Georgian (122 did not report ethnicity). The majority also reported spending their entire life in Turkey (*n* = 960). The average percentage of life spent in Turkey was 97.45 (*SD* = 12). The sample was diverse in religious affiliation. There were 93 atheists, 182 who believed in God but not a religion, 727 Muslims, and 71 affiliated with various other categories (Christians, Jews, Buddhists, agnostics; 134 did not report religious affiliation).

Throughout the survey, faces were displayed in 483 pixels (width) by 600 pixels (height)—dimensions chosen to fit in the screens of most devices. Each participant only dealt with 16 (8 male, 8 female) faces randomly chosen from the whole set of faces. Upon introducing the survey and obtaining informed consent, the randomly selected faces were displayed one at a time, blocked by gender and the order of gender determined randomly. In this first phase, participants merely indicated whether they recognized each face with a “yes” or “no” response.

Subsequently, participants rated each of these 16 faces on five dimensions, separately. The five dimensions were chosen to represent known sources of variance in face and social perception and with research interests of potential users of the database in mind: dominance, trustworthiness, attractiveness, masculinity, and femininity. These ratings were collected on the same verbally anchored Likert-type scale ranging from 1 to 7 (1: “not at all”; 7: “very much”). In addition, to obtain an estimate of how much each target looked like other people who participants encountered in their daily life in Turkish society (“Turkishness” for short), participants were asked to report how much each face looked like a person who was born/resides in Turkey on a 1 to 5 scale (1: “certainly a foreigner”; 2 “probably a foreigner”; 3: “I’m not sure”; 4: “probably born/resides in Turkey”; 5: “certainly born/resides in Turkey). We did not ask the simpler question of “how Turkish the face looks” because Turkish is used both as an ethnic label and the superordinate identity for people of different ethnicities sharing Turkish citizenship.

Throughout these ratings, participants were shown one face at a time and asked to rate the presented face on the presented dimension. The next face appeared on the screen after the participants clicked the “next” button (i.e., ratings were self-paced). To prevent participants from using their own previous rating(s) of a face as an anchor for subsequent ratings (i.e., if one gives a certain face a high rating on masculinity, one can give the same face a low rating on femininity without sufficient thought) or to apply their implicit personality theory to the ratings, and to reduce the demand to provide consistent ratings, we blocked the ratings by dimension. We also blocked the ratings by target gender because pilot testing revealed that raters subjectively experience using different standards for male and female targets and the task is experienced as more tiresome if target gender is randomly switched, even if the rating dimension remains constant. That is, target faces of the same gender alternated while the rating dimension remained in place until all the faces of that gender had been rated on that dimension. The order of dimension-by-gender blocks was randomized by the survey application so that each next block could be any combination of gender and dimension (e.g., male dominance, male trustworthiness, female attractiveness, male masculinity, etc.). The order of faces within each block was also randomized. When a dimension was finished, participants received a note that a new dimension was being introduced. Two other dimensions were collected for separate research purposes and are not reported here.

Because faces were selected randomly for each participant, the number of responses varies slightly for each face-by-rating instance (range: 48–84; on average, there were 66.43 responses per face-by-rating instance). The intraclass correlations (ICC) assessed the extent to which norming participants were consistent in their ratings of facial traits, separately for male and female faces. Because each face was rated by a random subset of perceivers, one-way, average-measures ICCs (i.e., ICC(1,k)) were computed [27]. These figures for male and female faces, respectively, were as follows: Attractiveness: .95, .96, Dominance: .95, .92, Femininity: .94, .95, Masculinity: .94, .94, Trustworthiness: .91, Turkishness: .94, .93. We provide the means and standard deviations for each face on each of these rating dimensions in the information file.

### Facial landmarks for Webmorph

Landmarks were placed on each facial photograph using Webmorph [28], a recent web-based version of Psychomorph. A total of 189 points were placed on each face, following sample templates (see http://users.aber.ac.uk/bpt/jpsychomorph/) and Sutherland’s [29] guideline. These are provided as separate files named “[photo ID number].tem” along with the photographs and they can be uploaded to Webmorph and used for performing various transformations such as averaging and morphing (see http://users.aber.ac.uk/bpt/jpsychomorph/).

### Facial landmarks for GM

We placed 72 landmarks on each facial photograph using tpsDig2 software, ver. 2.30 [30]. From total number of 72 landmarks, 36 should be treated as semilandmarks during analysis. Landmarks are corresponding locations that denotes homologous traits to which names could be given and which can be found in all specimens within a dataset. Semilandmarks (or sliders) are points located between landmarks that are used to denote curves or outlines where no true homologous traits could be unambiguously distinguished. Therefore, we provide supporting file in NTS format entitled as “sliders” and can be directly used within TPS-series of software for GM. The “sliders” NTS file contains 36 rows which denotes the number of semilandmarks and 3 columns wherein middle column gives the ID number of each semilandmark while the first and third columns provide the ID of the points between which the semilandmark is allowed to slid. We refer the reader to other sources for further definition of landmark and semilandmark locations on human faces [31,32]. We do not provide GM landmarks for images of the five females who wear the headscarf.

### fWHR

fWHR has drawn increasing research interest in the past decade. We provide this measurement for each facial photograph to facilitate research on this topic. Width and height measurements [33] were taken twice for each photograph by the same research assistant using NIH’s ImageJ (https://imagej.nih.gov/ij/) software. The two measurements were highly correlated (ICC *r* = .99 for width and *r* = .98 for height) and thus, they were averaged to obtain one width and one height measurement for each face. fWHR was subsequently computed by dividing width by height. It is difficult to obtain precise measurements for some photographs (e.g., facial features obscured by eyeglasses, headscarf, hair, etc.; head tilt, rotation, etc.). In these cases, approximations were made (e.g., position of cheekbones are estimated as closely as possible, when they are obscured). Thus, we caution researchers regarding the use of fWHR.

### Distance from average

Facial shape coordinates were superimposed by the generalized Procrustes analysis using the “gpagen” function implemented in the geomorph package in R [34]. This procedure converted all specimens to the origin, standardized the size of facial configurations, and optimized their rotation until the coordinates of corresponding points aligned as closely as possible. Semilandmarks were allowed to slide along tangents to a curve so as to minimize bending energy between each specimen and the Procrustes mean shape. The mean configuration (consensus) was computed separately for male and female photos. The “distance from average” (DFA) was computed as Procrustes distances between the mean shape and each configuration in the male or female set. Higher value indicates that a face is less close to the average).

### Maleness/Femaleness

To measure the individual degree of development of sexually dimorphic traits, i.e. morphological maleness/femaleness, we calculated a mean shape separately for male and female configurations. The position of an individual’s face along the axis connecting male and female mean shape then define its degree of geometric sexual dimorphism [35]. By projection of each individual on this axis, we obtained a score characterizing that individual’s facial maleness/femaleness. Higher positive scores indicate increasing maleness whereas higher negative scores indicate increasing femaleness.

## Results

Because the headscarf may obstruct parts of the face, making both fWHR measurement and GM landmark placement difficult, we report the results excluding the five females wearing the headscarf.

### Descriptive statistics for perceptual norms

**Fig 2:**
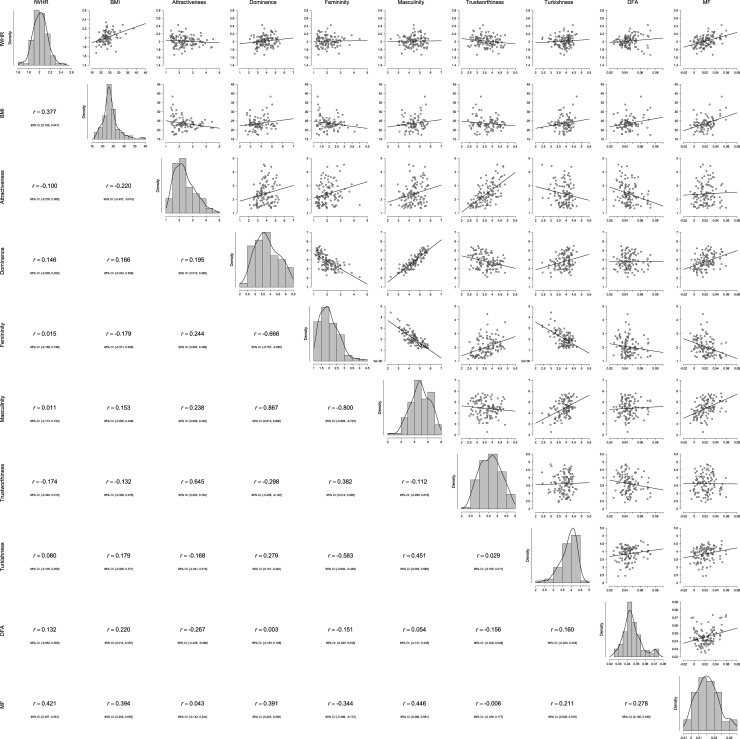
Histograms for and Correlations between fWHR, BMI, Perceptual Norms, and GM Measurements (DFA = Distance from Average; MF = Maleness/Femaleness) for Males.

**Fig 3:**
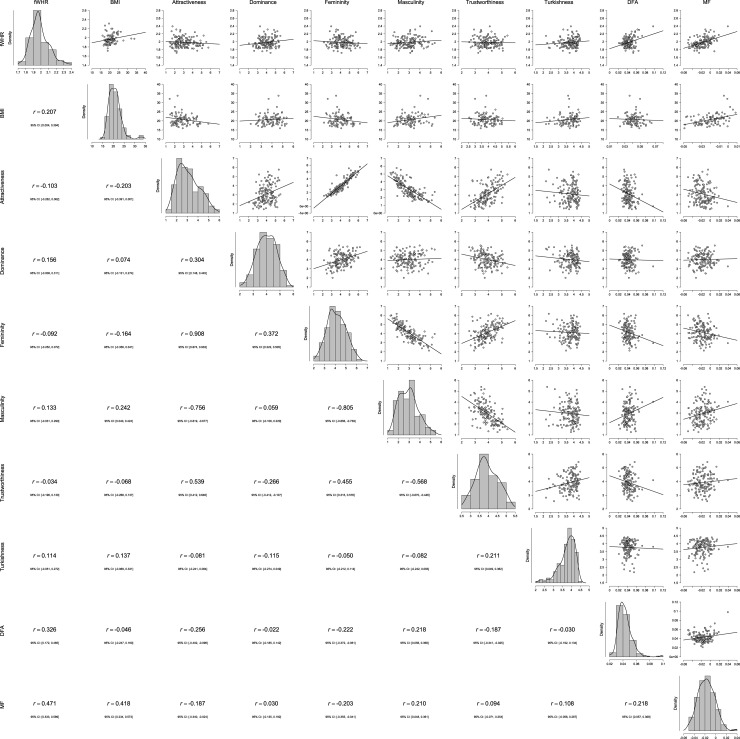
Histograms for and correlations between fWHR, BMI, perceptual norms, and GM measurements (DFA = Distance from Average; MF = Maleness/Femaleness) for Females.

Table 1 presents descriptives for the ratings of male and female faces by the norming sample, as well as fWHR, BMI based on self-reported height and weight, and GM measurements. Correlations among these variables and histograms, organized by gender of face-bearer, are shown in Figs 2 and 3. The pattern of correlations was largely consistent with the literature. For instance, consistent with the “what is beautiful is good” stereotype [36], more attractive faces tended to also be seen as more trustworthy. There were also unexpected effects. For instance, while the Turkishness of males was positively related to their perceived masculinity and dominance, the Turkishness of females was related positively only to their perceived trustworthiness.

**Table 1:** Descriptive statistics for fWHR, BMI, perceptual norms, and GM measurements.

Males										
	fWHR	BMI	Attractiveness	Dominance	Femininity	Masculinity	Trustworthiness	Turkishness	DFA	MF
Mean	2.017	23.64	2.403	3.788	2.031	4.429	3.612	3.858	0.045	0.022
SD	0.131	3.51	0.752	0.77	0.617	0.72	0.589	0.445	0.01	0.015
Minimum	1.667	17.36	1.342	2.108	1.164	2.274	2.419	2.429	0.025	-0.007
Maximum	2.426	38.31	4.539	5.478	4.425	5.789	4.84	4.684	0.073	0.06
Females										
	fWHR	BMI	Attractiveness	Dominance	Femininity	Masculinity	Trustworthiness	Turkishness	DFA	MF
Mean	1.985	20.75	3.096	4.003	4.124	2.946	3.925	3.761	0.043	-0.017
SD	0.118	2.92	1.003	0.72	0.846	0.886	0.617	0.456	0.01	0.015
Minimum	1.716	15.96	1.421	2.015	2.145	1.264	2.565	2.179	0.02	-0.05
Maximum	2.392	33.79	5.763	5.548	6.169	5.386	5.421	4.417	0.098	0.039

fWHR, Facial Width-to-Height Ratio; BMI, Body Mass Index; DFA, Distance from Average; MF, Maleness/Femaleness. Height and weight data were available for 91 males and 93 females.

### fWHR

There is an ongoing debate in the literature in terms of whether fWHR is sexually dimorphic. The early proposal that fWHR is linked to testosterone [37] suggests that male fWHR should be greater on average than female fWHR. There is mixed evidence on this issue [38]. Most relevant is Özener’s [39] finding of no dimorphism in a Turkish sample. An independent-samples t-test showed that, in our database, average male fWHR (*M* = 2.017, *SD* = 0.131) was indeed greater than average female fWHR (*M* = 1.985, *SD* = 0.118), *t*(257) = 2.076, *p* = 0.039, *Cohen’s d* = 0.26. However, Kramer [40] has shown that the sexual dimorphism on fWHR in his sample could be explained by body mass index (BMI). Thus, we calculated the body mass index using the formula BMI = (weight(kg) / height(cm)^2^)*10000, based on the subset of participants whose self-reported height and weight were available (91 males and 93 females). We conducted an ANCOVA with gender as the sole factor, fWHR as the dependent variable, and BMI as the sole covariate. BMI’s effect was significant, *F*(1,181) = 18.815, *p* < .001, *partial* η^*2*^ = 0.094; whereas gender had no main effect on fWHR after controlling for BMI, *F*(1,181) = 0.129, *p* = .72, *partial* η^*2*^ = 0.001. In conclusion, as in Kramer’s data, the sexual dimorphism present in this sample of faces was accounted for by BMI.

Figs 2 and 3 present the correlations between fWHR, BMI, ratings from the norming sample, and GM measurements, separately for male and female faces. For male faces, the pattern of correlations was consistent with the literature on fWHR, but none reached significance. For female faces, fWHR was related significantly and positively only to perceived dominance. Because of missing height and weight data, it is difficult to compare these correlations with partial correlations that control for BMI. However, partial correlations controlling for BMI do not result in substantively different figures for any of the pairs.

### GM of human face

**Fig 4:**
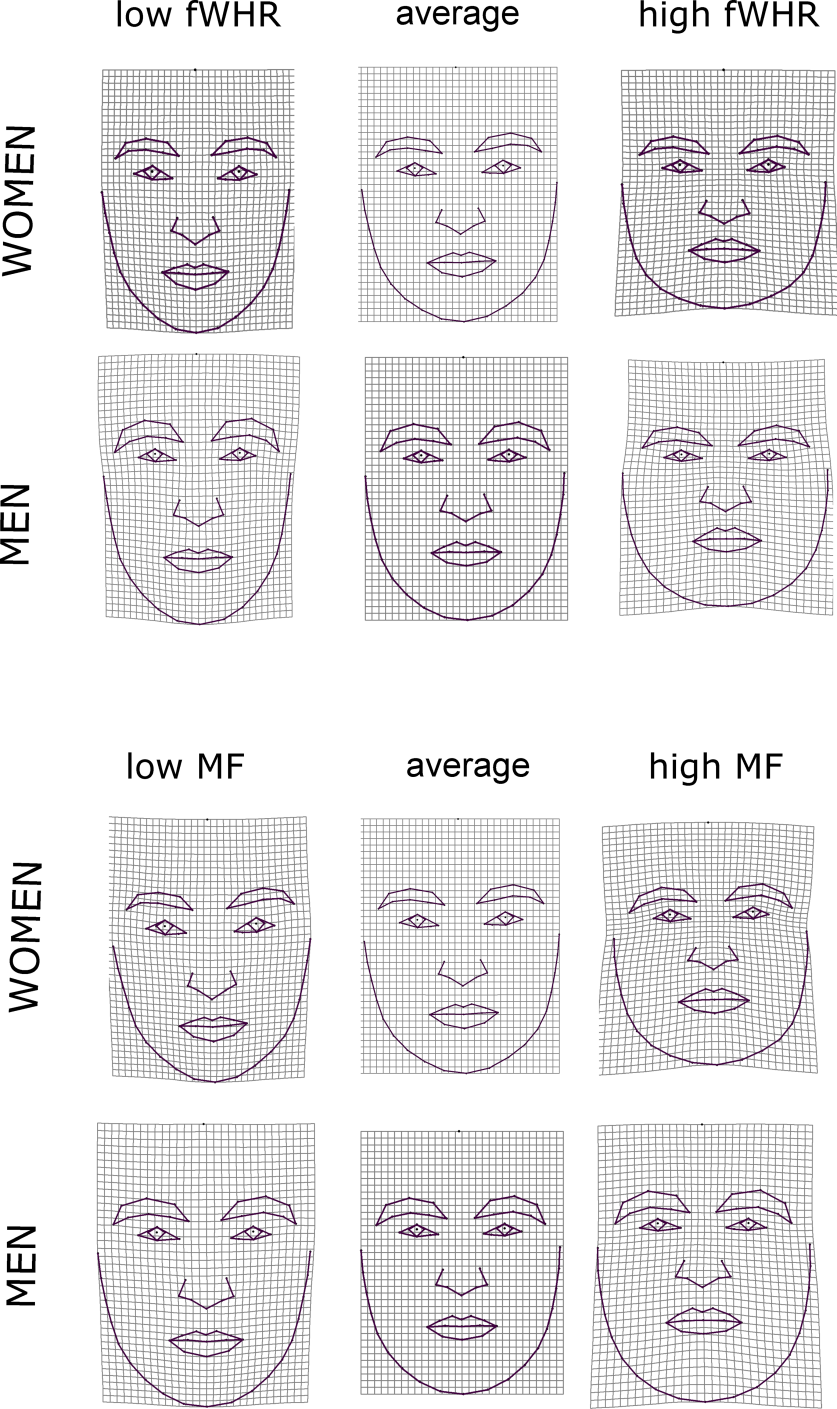
Thin plate-splines demonstrating the results of multivariate regression of shape coordinates on fWHR and scores of maleness/femaleness. Deformation grids shows differences in facial shape associated with high and low value of measurements for both men and women compared to an average configuration in the middle.

**Fig 5:**
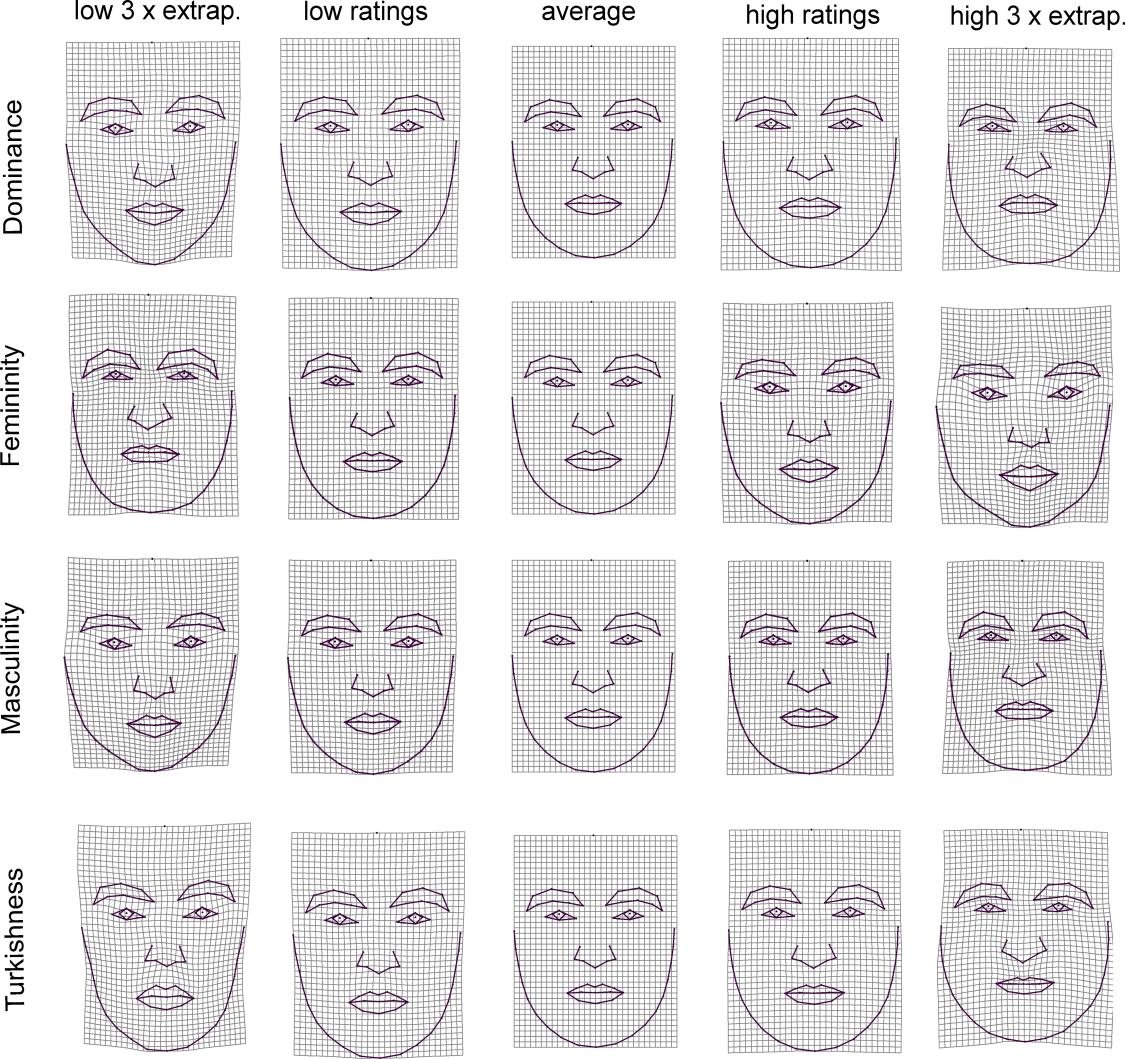
Visualization of shape regressions illustrating changes in facial shape associated with perception of dominance, femininity, masculinity, and Turkishness for males. Each perceived characteristic is shown as thin plate-spline deformations (within observed range and 3x extrapolated) compared to a consensus in the middle. The results for attractiveness and perceived trustworthiness were not statistically significant and cannot be visualized.

**Fig 6:**
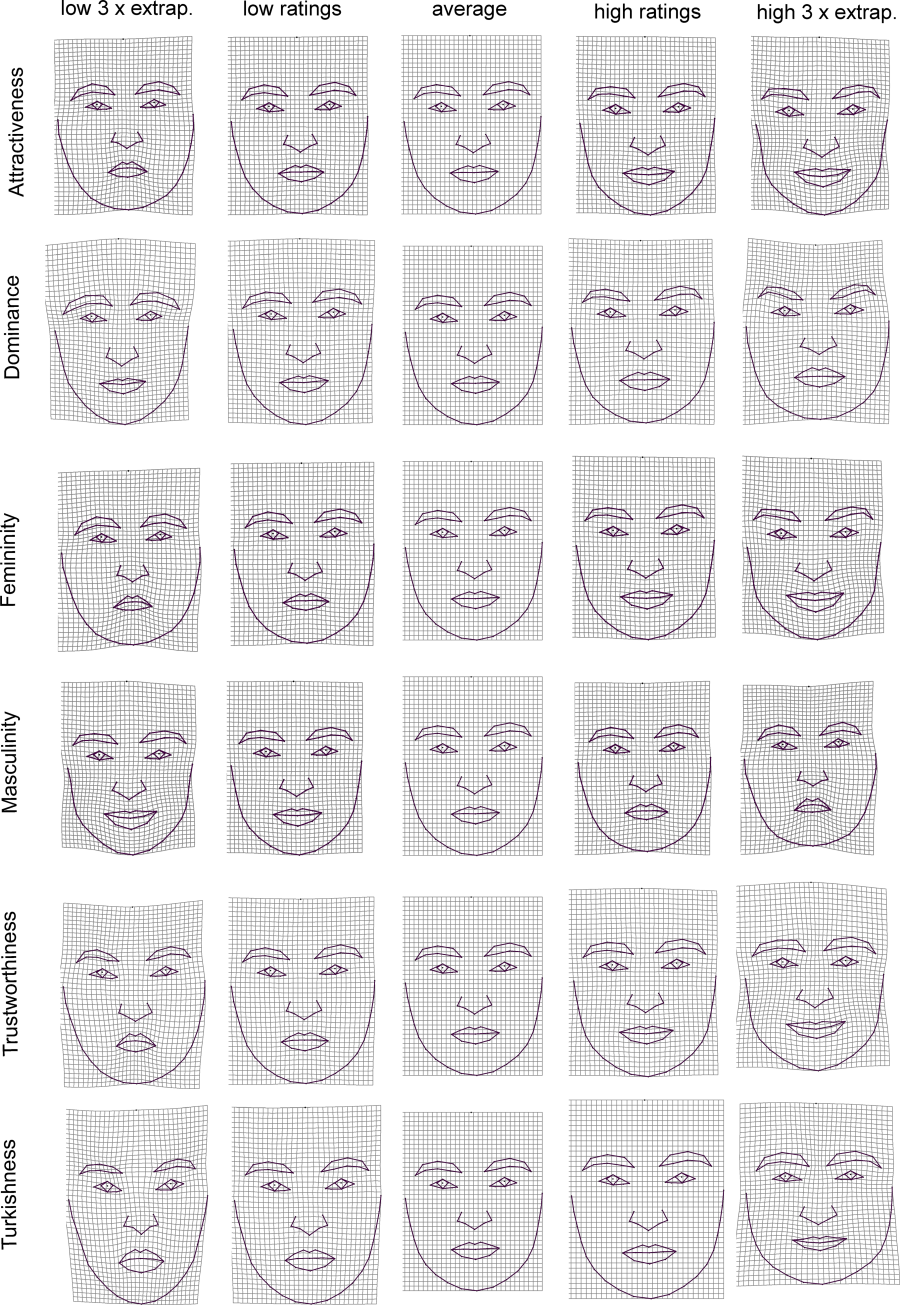
Visualization of shape regressions illustrating changes in facial shape associated with perception of attractiveness, dominance, femininity, masculinity, trustworthiness, and Turkishness for females. Each perceived characteristic is shown as thin plate-spline deformations (within observed range and 3x extrapolated) compared to an average configuration in the middle.

**Fig 7:**
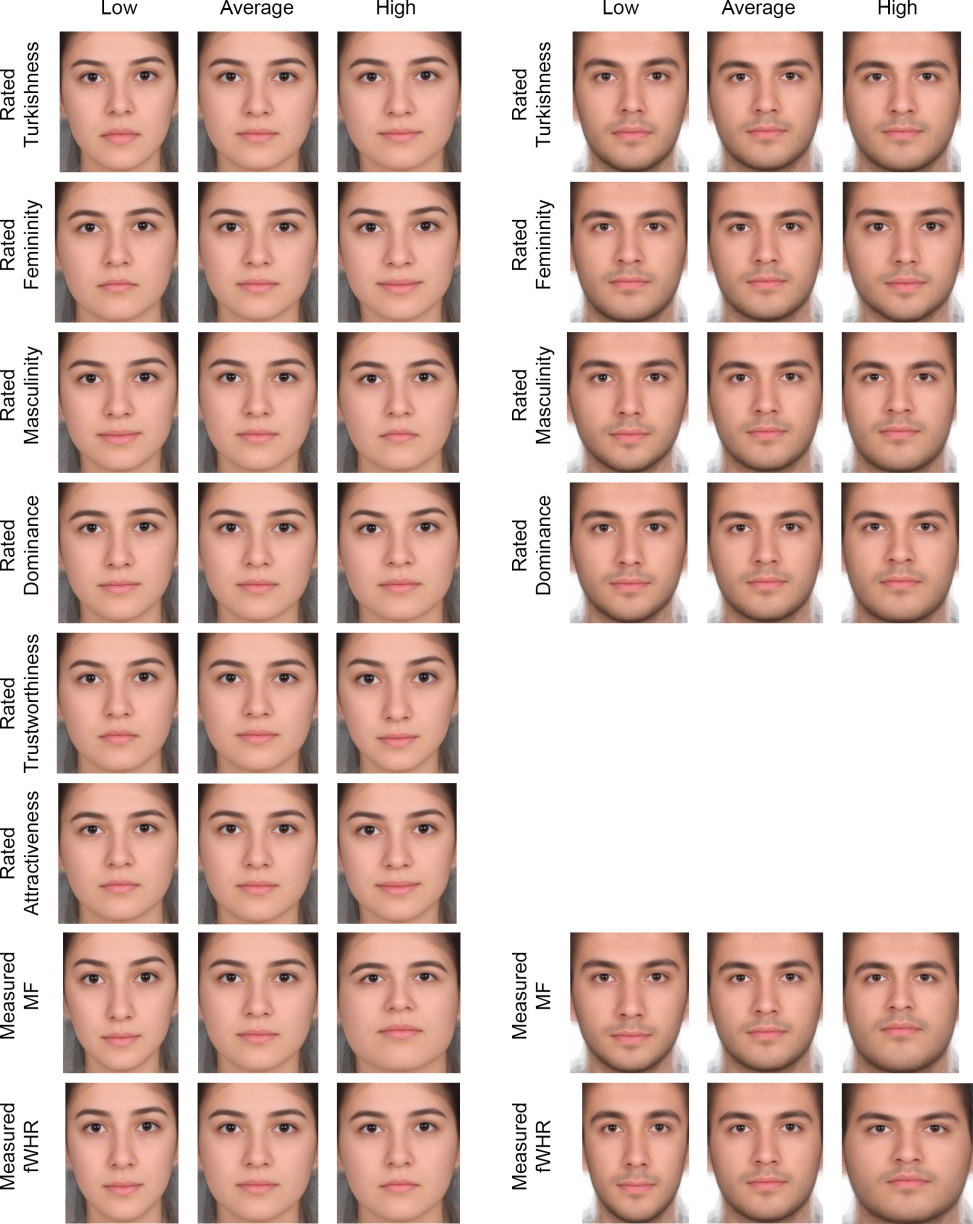
Facial composites demonstrating changes in facial shape associated with perception of attractiveness, dominance, femininity, masculinity, trustworthiness, Turkishness, fWHR, and scores of maleness/femaleness (MF) for females (left panel) and perception of dominance, femininity, masculinity, Turkishness, measures of fWHR, and scores of MF for males (right panel). Each perceived characteristic shows composite consisting of 10 averaged facial textures unwarped to predicted configuration (within observed range) compared to a consensus in the middle. The results for attractiveness and perceived trustworthiness in men were not statistically significant and cannot be visualized.

We employ the regressions of Cartesian shape coordinates on particular ratings and measurements to explore how these predictors are related to the variation of facial shape. The shape variation predicted by ratings of attractiveness, dominance, femininity, masculinity, trustworthiness, and Turkishness as well as shape changes associated with fWHR and geometric measure of maleness/femaleness (MF) were visualized via thin-plate spline deformation grids as deviations from the overall mean configuration (consensus) of landmarks (Figs 4–7). The test statistics and effect sizes are summarized in Table 2.

**Table 2:** Summary of results for shape regressions of facial coordinates on eight predictors.

	WOMEN			MEN		
	F	R-squared	p-value	F	R-squared	p-value
Attractiveness	3.44	0.023	0.0026	0.874	0.008	0.54
Dominance	2.016	0.014	0.043	2.54	0.022	0.01
Femininity	3.57	0.024	0.0017	1.932	0.017	0.0405
Masculinity	4.3	0.029	0.0004	2.774	0.024	0.0055
Trustworthiness	4.69	0.032	0.0002	0.008	0.921	0.485
Turkishness	5.34	0.036	0.0001	2.644	0.023	0.0092
fWHR	28.65	0.17	0.0001	22.47	0.165	0.0001
MF	25.56	0.152	0.0001	17.38	0.132	0.0001

## Discussion

The present effort attempted to produce a database of facial photographs and accompanying information and materials for use in scientific research. Much of the available facial stimuli sets are from Western countries. Only limited research has been carried out outside western, educated, industrialized, rich, and democratic (WEIRD) societies. The conclusions based on investigations in WEIRD societies could seriously bias our knowledge of human psychology as most of the people on the planet are not WEIRD [41]. Modern Turkish society as a successors of various Anatolian cultures combines Western secular trends with traditional societal values of Orient. Compared to U.S. and European standards, Turkish society shows greater intensity of inter-personal interactions [42]. Note also that the vast majority of current research is based on photographic data from Western countries and East Asia whereas data from Middle-East populations are sparse. Our current aim was to increase the range of accessible photographic materials. Specifically, we aimed to provide a set of stimuli from Turkey, whose population shows considerable variation in ethnic background and lifestyle preferences, reflected in the appearances of our targets. To increase the utility of our stimuli set, we provided norms from a large sample for how each target is seen on several widely researched traits. We also provided additional information that should aid researchers in selecting among these faces, as well as a recently popular facial measurement (fWHR) that has been linked to various perceptions and traits. Further, we provided facial landmarks that could be used to transform these faces in a freely available, state-of-the-art web application as well as landmarks for use in geometric morphometric analyses. Finally, we present basic correlational analyses (see Figs 2 and 3) and shape regressions to inspect the variation of facial shape associated with target perceptions and facial measures (see Figs 4–7). In general, the shape changes associated with higher attractiveness, trustworthiness, femininity, lower dominance, and lower masculinity converge in female faces. The faces of men exhibited a similar pattern. Interestingly, the shape variation characterized by Turkishness correlates with variation predicted by higher fWHR in both sexes and higher maleness in male faces. In face of women, Turkishness is also correlated with higher facial attractiveness. We believe that these descriptive results may be helpful for comparative purposes as well as for manipulation of composite facial images.

We modeled our effort partly after an excellent, recently published Western database [25]. However, our database of faces unfortunately did not include emotional expressions or people from other age groups. In addition, we did not have the means to restrict variation in clothing, make-up, facial decoration, and facial hair. This general limitation could also be an advantage in research contexts where more natural or diverse facial stimuli are required. For instance, the headscarf is a decorative cue to religious identity and our photographs of women wearing headscarves could be used in studies of prejudice [43]. Researchers should also exercise caution regarding more subtle variation in emotional expression and head position. For most research purposes, it should be possible to select a subset from these 264 photographs that are sufficiently constrained on chosen dimensions. Because there is only one image per face, the database is not suitable for use in face recognition research.

Because of our current interest in the fWHR specifically, we provided this facial metric but did not take other facial measures. However, as far as our knowledge goes, ours is the first facial database accompanied by geometric morphometric landmarks and these can be used readily to generate a variety of facial metrics. Users of our database are welcome to contribute by providing facial metrics other than fWHR, as well as any other supporting materials that they think will enrich the database.

The entire set of facial photographs, data, and supporting materials is available free of charge from the Department of Psychology at Bogazici University, for scientific research purposes to researchers affiliated with a higher education or research institution, after returning the signed agreement form (see S1 File). Researchers are kindly requested to send the completed agreement form to psy@boun.edu.tr (preferably using their institutional e-mail account to facilitate the process) in order to obtain the database.

## Supporting information

S1 File BFD v1 agreement form. The form that must be signed for requesting access to the database.
(PDF)
