# Supplementary material for: The Bogazici face database: Standardized photographs of Turkish faces with supporting materials
Source: PLoS One. 2018 Feb 14;13(2):e0192018. doi: 10.1371/journal.pone.0192018 (PMC5812588; doi:10.1371/journal.pone.0192018)
Supplement: S1 File — The form that must be signed for requesting access to the database. (PDF) [file pone.0192018.s001.pdf]

BOGAZICI FACE DATABASE
Version 1.0 – January 2018

The Bogazici Face Database is reported in detail in the following manuscript:

Saribay, S. A., Biten, A. F., Meral, E. O., Aldan, P., Třebický, V., & Kleisner, K. (in press). The Bogazici face database: Standardized photographs of Turkish faces with supporting materials. *Plos One.*

For all inquiries, address correspondence to:

Bogazici University, Department of Psychology
Bebek, Istanbul, 34342, TURKEY
e-mail: psy@boun.edu.tr
telephone: +902123596757

Terms of Use

By applying for permission to access the database, you agree to the following terms of use:

1. Upon completing the form below, the user is given permission to use the database for non-commercial, scientific research purposes only. Only users affiliated with a recognized higher education or research institution and who apply using a verified e-mail address from their institution will be given access to the database.
2. The identity of the photographed individuals is protected. The user is not allowed to attempt to identify the individuals whose photographs are included in the database.
3. Any scientific presentations (i.e., conference poster, manuscript, thesis, dissertation, etc.) of research making use of (i.e., reporting data based on) the database must acknowledge their source (i.e., the citation above).
4. The database may not be (re-)distributed individually or in bulk under any circumstances, in original or modified form. For permission to display the photographs in a research manuscript or scientific presentation, please send an e-mail to the author (see above).
5. Bogazici University and the authors of the database are not responsible for any damage that may be caused by the use of the Bogazici Face Database.

Request for Permission to Use the Bogazici Face Database:

"I consent to all of the terms of use stated in this document regarding the Bogazici Face Database."

Full name and title: ______________________________ Signature: ______________________________

Date of Request: _____________________________

Current Institution: _____________________________________

E-mail: __________________________________

Please complete this form electronically (with your signature) or scan after completing a printed form and send it in an e-mail to psy@boun.edu.tr

Please consider contributing to the database by reporting mistakes and by providing any materials and measurements you generate based on the included photographs.
